# Editorial: COVID-19 and human reproduction

**DOI:** 10.3389/frph.2023.1202180

**Published:** 2023-04-26

**Authors:** Jan Tesarik

**Affiliations:** MARGen Clinic, Molecular Assisted Reproduction and Genetics, Granada, Spain

**Keywords:** COVID-19, human reproduction, assisted reproduction, sperm quality, oocyte quality, pregnancy outcomes, vaccination effects.

**Editorial on the Research Topic** COVID-19 and human reproduction

At the time COVID-19 was declared as pandemic by the World Health Organization (WHO), on 11 March 2020, much insecurity was created among the patients and doctors involved in the planning and performing assisted reproduction treatment (ART) (1). At that time, scientific data concerning potential risk of COVID-19 in ART were scarce, and sometimes controversial. As a matter of example, one study reported various kinds of serious health problems in nine out of ten neonates born to mothers with pneumonia caused by SARS-CoV-2, the virus responsible for COVID-19 disease (2), another study reported the birth of nine normal children to mothers with laboratory-confirmed COVID-19 (3). Because of the lack of solid evidence showing negative effects of COVID-19 on ART outcomes and human reproduction in general, and in view of the fact that each year may count for the success or failure of ART in some infertile couples, I suggested ART attempts not to be discontinued in the COVID-19 era (1).

Subsequent reports mostly backed up this recommendation. In order to update and clear up further this subject, the Journal *Frontiers in Reproductive Health* launched a special Research Topic “COVID-19 and Human Reproduction”. In this Editorial, I resume the main points presented in the articles published in this article collection. These articles focus on sperm, oocyte and embryo quality, as well as pregnancy outcomes, in persons who suffered the infection or underwent anti-SARS-CoV-2 vaccination, in addition to the consequences of the pandemic on general problems related to the sexual and reproductive health.

In their Original Research paper, Stigliani et al. investigated into possible long-term effects of COVID-19 disease on sperm parameters. This longitudinal retrospective study evaluated sperm parameters collected from 20 men before and after infection, in addition to comparing the reproductive potential in pre- and post-COVID-19 infertility treatments of 8 self-controlled couples as well as in 40 cycles after COVID-19 infection of the male partner. Data obtained are reassuring in that COVID-19 disease has no negative effects on sperm quality and male reproductive potential when semen samples are collected three months or more after infection.

Another Original Research article, authored by Huri et al. assessed the impact of COVID-19 pandemic on the outcomes of ART pregnancies, taking into account the occurrence of early pregnancy loss, overall success rate, and live birth rate. This single-center retro-prospective cohort study compared ART outcomes in 791 patients who underwent ART treatments from 1 March 2020 to 28 February 2021 (pandemic ART cohort) with those obtained in 844 patients treated from 1 March 2019 to 29 February 2020 (control cohort). No statistically significant differences in implantation rate, clinical pregnancy rate, ectopic pregnancy rate, the first and second trimester miscarriage rate, and live birth rate were detected between the two cohorts. The authors conclude that the COVID-19 pandemic did not cause any significant changes in ART pregnancy outcomes, and patients and physicians should be reassured in this sense.

Despite the fact that COVID-19 vaccination protects against potentially serious consequences of SARS-CoV-2 infection, some people have been hesitant to receive the vaccine because of fears about its negative effects on future menstrual cycles. In order to assess this possible risk, Alvergne et al. published an Original Research article reporting results of a combined prospective/retrospective study evaluating the effects of COVID-19 vaccination on the timing and flow of menstrual period. Altogether, the data reported show that COVID-19 vaccination is not associated with any middle- or long-lasting perturbations of menstrual flow in healthy women, irrespectively of the brand of vaccine used. However, the data also suggest that individuals with endometriosis or polycystic ovary syndrome may be more likely to notice a change in their periods, the subject that warrants further investigation.

Even though either a past COVID-19 infection or COVID-19 vaccination are highly unlikely to affect human fertility directly, there might be indirect effects on sexual and reproductive health, relating to the functioning and occupation of health services, and such indirect effects might be more likely to occur in underdeveloped countries. In this sense, an Original Research article by Mambo et al. shows how the access to sexual and reproductive health services was affected by COVID-19 pandemic in Uganda. They conclude that the access to sexual and reproductive health (SRH) information and services was restricted in Uganda during the COVID-19 lockdown because of the lack of transportation, long distances to health facilities, and high cost of services, leading to an increase in various SHR risks and adverse outcomes.

In conclusion, data presented in this article collection are reassuring because no long-lasting negative effects of previous COVID-19 infection or COVID-19 vaccination could be demonstrated. However, indirect effects on general SRH, related to limitations of the access to health services, may occur, and this should be taken into account for planning a better preparation for case of future unexpected health calamities.
